# Supplementary material for: Activation of Interferon-Stimulated Genes following Varicella-Zoster Virus Infection in a Human iPSC-Derived Neuronal In Vitro Model Depends on Exogenous Interferon-α
Source: Viruses. 2022 Nov 14;14(11):2517. doi: 10.3390/v14112517 (PMC9693540; doi:10.3390/v14112517)
Supplement: Supplementary file 1 [file viruses-14-02517-s001.zip › viruses-1939206-supplementary.pdf]

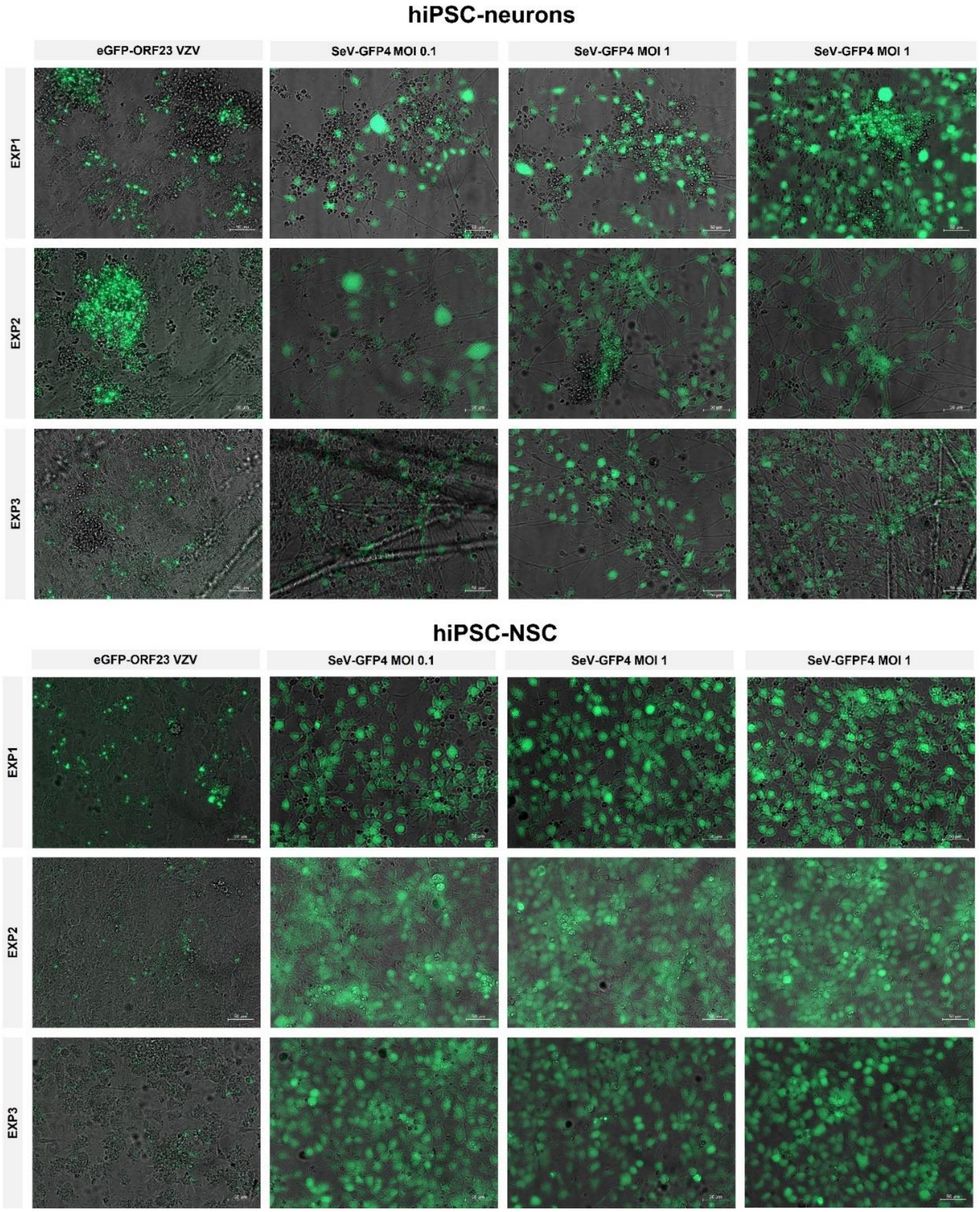

Supplementary Figure S1: Immunofluorescent images of hiPSC-neurons and hiPSC-NSC 24h post-inoculation with eGFP-ORF23 VZV lysate or SeV-GFP. Representative immunofluorescent images at 24hpi of hiPSC-neurons (upper panels) and hiPSC-NSC (lower panels) from each experiment, inoculated with $1.5x10^2$ PFU eGFP-ORF23 VZV lysate or SeV-GFP at MOI 0.1, 1, or 10 are shown, indicating successful infection by expression of eGFP and GFP, respectively.
